# Highly Stable Wideband Microwave Extraction by Synchronizing Widely Tunable Optoelectronic Oscillator with Optical Frequency Comb

**DOI:** 10.1038/srep03509

**Published:** 2013-12-16

**Authors:** D. Hou, X. P. Xie, Y. L. Zhang, J. T. Wu, Z. Y. Chen, J. Y. Zhao

**Affiliations:** 1 Department of Electronics, Peking University, Beijing 100871, China, State Key Laboratory of Advanced Optical Communication Systems and Networks, Peking University, Beijing 100871, China

## Abstract

Optical frequency combs (OFCs), based on mode-locked lasers (MLLs), have attracted considerable attention in many fields over recent years. Among the applications of OFCs, one of the most challenging works is the extraction of a highly stable microwave with low phase noise. Many synchronisation schemes have been exploited to synchronise an electronic oscillator with the pulse train from a MLL, helping to extract an ultra-stable microwave. Here, we demonstrate novel wideband microwave extraction from a stable OFC by synchronising a single widely tunable optoelectronic oscillator (OEO) with an OFC at different harmonic frequencies, using an optical phase detection technique. The tunable range of the proposed microwave extraction extends from 2 GHz to 4 GHz, and in a long-term synchronisation experiment over 12 hours, the proposed synchronisation scheme provided a rms timing drift of 18 fs and frequency instabilities at 1.2 × 10^−15^/1 s and 2.2 × 10^−18^/10000 s.

An optical frequency comb (OFC), a light source whose spectrum consists of a series of discrete, equally spaced elements, can generate femtosecond pulse trains with low timing jitter^1^. OFCs are powerful tools for establishing a link from radio frequency standards to optical frequencies and have been widely used in many fields^2^,^3^,^4^,^5^, for example, in frequency metrology, timing distribution, optical communication, and optical clocks. There is considerable interest in achieving a highly stable and low-noise microwave source from a stabilised OFC^6^. High-quality microwaves could improve the performance of radar systems^7^, increase the resolution of very-long baseline interferometry detection^8^, and enhance precision in the synthesis of microwave frequencies^9^. As the purest microwave sources, OFCs naturally have many harmonics with very low short-term phase noise^10^,^11^.

Recently, some new stabilisation techniques were developed to lock OFCs to cavity-stabilised lasers for the generation of ultra-stable pulse trains^5^,^6^,^12^. Due to the ultra-low noise and ultra-high stability of the stabilised OFCs, it has been anticipated that OFCs would synchronise large-scale scientific facilities requiring extremely high timing accuracy^13^,^14^,^15^. In practice, synchronised facilities usually include low phase noise performance voltage-controlled oscillators (VCOs). VCOs generally exhibit very low short-term phase noise. However, as the carrier frequency of the VCO increases, the phase noise performance deteriorates^16^. Therefore, when the VCO oscillates at a higher frequency, its short-term phase noise will increase. In addition to this disadvantage of noise deterioration, the tunable range of low-noise VCOs is usually very small. For example, a conventional dielectric resonator oscillator (DRO)^17^, a typical high-frequency VCO, oscillating at 9.56 GHz has a tunable bandwidth of +/−20 MHz. In practical extraction applications, we have to use different VCOs with various frequencies, if the different harmonics of the OFC will be extracted.

Compared to a conventional VCO, an optoelectronic oscillator (OEO) with a long delay fibre link can generate a high-purity microwave with the same low phase noise at a very high carrier frequency^18^,^19^,^20^. With the advantage of low phase noise at any oscillation frequency, OEOs can replace most electronic oscillators to obtain ultra-pure microwaves, especially at the high-frequency bands of several or tens of GHz; for example, a compact 10-GHz OEO has been reported^21^ with an ultra-low phase noise of −163 dBc/Hz at 6 kHz offset frequency. In addition, it is easy to implement a wideband OEO through a tunable electronic or photonic filter^22^. Therefore, we can synchronise a single wideband OEO with the harmonics of a stabilised OFC to realise wideband extraction of the OFC. In this study, we report a scheme for synchronising a frequency-stabilised OFC with a tunable OEO rather than a VCO. This synchronisation approach can extract a highly stable and wideband microwave with very low phase noise, and in this approach, the tunable OEO is locked to the harmonics of the OFC at arbitrary harmonic frequencies.

In this synchronisation approach, photodetection and phase detection are two absolutely necessary techniques. Many reports have revealed that excess phase noise is added in the optical-to-electronic (O/E) conversion process due to nonlinearity, saturation, temperature drift, and amplitude-to-phase conversion in photodiodes when direct photodetection is used^23^. Therefore, we utilised a fibre-based optical-microwave phase detector^15^ between the OFC and OEO to implement a phase-locking loop (PLL), achieving synchronisation with ultra-low residual phase noise and very-low long-term phase drift. Our microwave synchronisation system, illustrated in Figure 1, achieves extraction of wideband microwaves with the same purity (phase noise) and long-term high stability at an arbitrary harmonic frequency, where the frequency of the extracted microwave is selected by a tunable electronic filter with a narrow bandwidth. Due to the very low phase noise of tunable OEOs across the entire tunable frequency range, the synchronisation of an OFC to an OEO is an advisable way that combines the advantages of the OFC and OEO in the extraction of a high-purity and stable wideband microwave. To our knowledge, this is the first report of wideband frequency extraction from an optical pulse train with a single oscillator.

## Results

### Widely tunable OEO

OEOs are based on converting the continuous light energy from a pump laser to radio frequency (RF) and microwave signals. OEOs are characterised by a very high purity, as well as other functional characteristics that are not readily achieved with electronic oscillators. The unique behaviour of the OEO benefits from the use of an electro-optical modulator (EOM), a long delay fibre, and other optical components that are generally characterised by a very high quality factor (Q), high efficiency, high speed, and low dispersion in the microwave frequency regime.

In this study, we designed a widely tunable OEO and synchronised it with the pulse train of a stable OFC to extract a series of highly stable microwaves with very low phase noise. This OEO has a tunable oscillation frequency range of 2 GHz to 4 GHz. Figure 2 shows a schematic of the proposed tunable OEO. Light waves from a DFB laser, with a tunable wavelength of 1545 nm to 1555 nm and an average optical power of approximately 10 mW, are sent to an EOM that is amplitude-modulated by the microwave feedback signal. The modulated light wave generated by the EOM is then launched to a 500-m dispersion-decreasing fibre (DDF). At the end of the fibre, a fast PIN photodetector converts the modulated light to a microwave signal with a series of side modes. The microwave is amplified by a low-noise electronic amplifier and enters a tunable bandpass filter (BPF) group consisting of two tunable BPFs (Hittite, HMC89LP5E). The filter group can attain a very high Q value through adjustments in the bandwidths of two filters. If the bandwidth of the filter group is narrow enough to filter only one of the first-order side modes of the microwave, a pure microwave with low-intensity side modes is obtained. The filtered microwave is amplified by a high-gain amplifier and is then split into two parts by an electronic coupler (90:10). The higher-powered portion is fed back to the EOM to create the OEO, and the lower-powered part, with a power of approximately 3 dBm, is used for the output. The frequency of the OEO can be coarsely adjusted by tuning the centre frequency of the filter group and can be finely adjusted by tuning the wavelength of the DFB laser source. With the flexible tuning, it is easy to make the OEO oscillate at a frequency that is very close to a harmonic of the OFC.

### Experimental setup and operation

The setup of the microwave extraction by synchronizing OEO with the OFC is illustrated in Figure 2. The setup contains an optical phase detector, phase-locking circuits, an OEO with a tunable frequency range of 2 GHz to 4 GHz, and an Er-doped fibre-based OFC with a fundamental repetition rate of 144.5 MHz. The OFC is stabilised to an H-master clock (the details of the stable OFC will be described in the Methods section). The objective of the synchronisation system is to establish a phase-locking loop between the widely tunable OEO and the stable pulse trains of the OFC through optical phase detection. The pulse train generated from the H-master-stabilised OFC with an average optical power of approximately 35 mW is split and applied to two independent optical phase detectors. The microwave signal from the OEO is also split into two parts with a power splitter. These two parts are both amplified to approximately 16 dBm and are then used to compare the pulse trains in the optical phase detectors to generate two phase errors. One phase error is low-pass filtered, amplified, (Proportion Integration Differentiation) PID-regulated, and fed back into the voltage-bias port of the OEO to phase-lock the OEO at a 10-kHz locking bandwidth. The other phase error signal is sent to a signal analyser and a data recorder for out-of-loop measurements. In addition, a phase noise analyser monitors the out-of-loop microwave to acquire the absolute single-sideband (SSB) phase noise.

### Frequency instability and phase noise

To investigate the performance of the extraction scheme, we conducted a long-term synchronisation experiment. To create a stable environment, the OFC, OEO, and optical phase detector were individually sealed in closed containers. Active temperature controllers were used to monitor and stabilise the temperatures of these containers. To avoid air flow and acoustic vibration, the containers were surrounded by vibration-isolation foam with a thickness of approximately 20 mm. With this environmental protection, most effects introduced by temperature fluctuations and air flow were reduced. By tuning the centre frequency of the tunable filter, a widely tunable OEO range of 2 to 4 GHz was achieved with a frequency step of 144.5 MHz (the repetition rate of the OFC). In Figure 3, we present the spectra of the microwaves generated from the free-running OEO at eleven discrete frequencies, from the 16^th^ to the 26^th^ harmonic of the OFC (2.312, 2.457, 2.601, 2.746, 2.890, 3.035, 3.179, 3.324, 3.468, 3.613, and 3.757 GHz). In our synchronisation experiment, the microwaves generated from the tunable OEO at these eleven discrete frequencies were synchronised with the eleven harmonics (16^th^–26^th^) of the pulse train.

Figure 4a shows the SSB phase noise measurements obtained at a carrier frequency of 3.035 GHz (21^st^ harmonic): curves (i) and (ii) show the absolute phase noise of the free-running OEO and the locked OEO, respectively. The phase noise of the free-running OEO is greater than −40 dBc/Hz at 1 Hz offset and approximately −140 dBc/Hz at 100 kHz offset. Compared with the free-running OEO, the optical phase-locking loop improves the phase noise to approximately −75 dBc/Hz at 1 Hz offset. Curve (iii) shows the phase noise of a commercial free-running VCO (Hittite, HMC416LP4). The phase noise of the free-running OEO is clearly lower than that of the commercial VCO at the measured offset frequencies of 10 Hz to 100 kHz. Curve (iv) shows that the out-of-loop residual phase noise of the locked OEO reaches −113 dBc/Hz and −143 dBc/Hz at 1 Hz and 100 kHz offset frequencies, respectively; this residual phase noise also results in a 2.2-fs rms timing jitter integrated from 1 Hz to 100 kHz. Curve (v) displays the background noise of the optical phase detector when only the pulse train is applied to the optical phase detector without a microwave signal. The experimental phase noise results demonstrate that the synchronisation scheme using the optical phase detector has good locking performance. In addition, we measured the absolute SSB phase noise of the locked OEO at the eleven discrete carrier frequencies. The absolute SSB phase noise measured at offset frequencies of 0.1, 1, 10, and 100 kHz, shown in Figure 4b, demonstrates that the OEO has approximately same low phase noise throughout the entire tunable carrier frequency range.

Figure 5a shows the phase drift between the pulse train and the synchronised OEO at 3.035 GHz, sampled at 2 samples/s with a 100-Hz low-pass filter. The figure shows that the phase drift curve is almost flat over the measurement time (12 hours), and the integrated phase drift is 18 fs (rms). Based on the measured phase drift, we also calculated the fractional frequency instability. Figure 5b shows that the relative frequency instability of the synchronisation loop between the OEO and the OFC is 1.2 × 10^−15^ for an averaging time of 1 s and 2.2 × 10^−19^ for an averaging time of 10000 s (filled triangles in Fig. 5b). This ultra-low instability curve implies that stability has been transferred from the stable OFC to the locked OEO without stability loss. In addition, the instabilities of the H-master-stabilised OFC (filled squares in Fig. 5b) and the optical clock^5^ (filled circles in Fig. 5b) are also demonstrated in the same figure.

## Discussion

The proposed wideband microwave extraction from optical pulse trains, in which a tunable OEO is directly synchronised to an OFC through the optical-microwave phase detection technique, differs from existing microwave extraction techniques. In previously reported extraction systems^12^,^13^,^14^,^15^, the microwave generated from a VCO is usually phase-detected with a pulse train and produces a phase-error signal at the intermediate frequency (IF) port. This error signal is low-pass filtered, amplified, PID-adjusted, and then fed back into the VCO to synchronise the VCO with the pulse train. In this case, the VCO, with its narrow tunable bandwidth, can be only synchronised with one of the harmonics. This result implies that different VCOs must be used if we want to extract different harmonics of the pulse train. In addition, a higher-frequency VCO usually introduces greater SSB phase noise. Figure 4a shows that the commercial VCO has a higher phase noise than our OEO at low and intermediate offset frequencies in the free-running state. These disadvantages of the VCO will limit its use in practical applications. Therefore, to avoid these limitations, a widely tunable OEO is introduced to replace the conventional electronic VCO. The proposed extraction approach, which utilises the characteristics of the OEO as described in the previous section, can extract a series of harmonics of the pulse train through synchronisation between a single widely tunable OEO and the pulse train. This extraction is difficult to achieve with a single electronic oscillator. This advantage allows the OEO to synchronise with the OFC over a very wide frequency range, e.g., tens of GHz.

After synchronisation is established, the residual phase noise is a key parameter in determining the short-term synchronisation performance of our extraction system. Therefore, we measured the out-of-loop residual phase noise between the synchronised OEO and the OFC at a carrier frequency of 3.035 GHz. We believe that the decent residual phase noise of −113 dBc/Hz and −143 dBc/Hz at 1 Hz and 100 kHz offset frequencies, respectively, is sufficient to support the excellent short-term synchronisation performance provided by the optical phase-locking system. The residual phase noise in the locking bandwidth (<10 kHz) is limited by the noise of the synchronisation system, and the noise outside the locking bandwidth (>10 kHz) is limited by the natural phase noise of the free-running OEO, respectively. The absolute phase noise of the OEO in our synchronisation system is not superior to that of the VCOs reported by Fortier et al. and Jung and Kim [12, 15]. We believe this result occurred because the elements used in our OEO loop are commercial products, which would limit the phase noise performance. In particular, the tunable filter, whose noise is related to the tunable range, is the crucial factor determining the phase noise level. Although the phase noise of our OEO has these limitations, the wide tuneability of the microwave extraction is our primary focus and represents a significant advantage. In future work, we will develop a lower phase noise OEO at a similar or wider tunable range level.

The frequency instability is another key parameter in determining the long-term synchronisation performance of our extraction system. Therefore, we measured the timing drift and the relative frequency instabilities of the synchronisation loop between the OEO and OFC. The 18-fs integrated timing drift (rms) is larger than the value reported in Jung and Kim^15^. We believe that this result occurred because the OEO, with its long delay fibre, occupies a large space. In this case, the effects of temperature fluctuation and air flow cannot be completely eliminated in such long time of 12 hour. Figure 5b shows that the frequency instability of the locked loop is 1.2 × 10^−15^ for an averaging time of 1 s and 2.2 × 10^−18^ for an averaging time 10000 s; this frequency instability is much lower than that of the stabilised OFC shown in Figure 5b. Thus, the stability of the OFC will be directly transferred to the OEO without any stability loss. Furthermore, the instability of the locked loop in our synchronisation system is even lower than that of an optical clock. This result implies that the proposed synchronisation technique can be used to synchronise a tunable OEO with an optical clock signal, that is, to transfer the stability of the optical clock to the OEO and achieve a wideband synchronisation of optical clock signal.

Finally, we note that the frequency of the microwave at 3.035 GHz (21^st^ harmonic) in our measurement results was not deliberately considered, but arbitrarily chosen. Any microwaves at harmonic frequencies within the tuning range of the OEO would exhibit the same experimental results in our synchronisation system. The frequency range of 2 to 4 GHz in our experiment is limited by the electronic filter, and a wider frequency range could be achieved by utilising a tunable filter with a wider tuning range.

## Methods

### Optical phase detector

**Figure 1:**
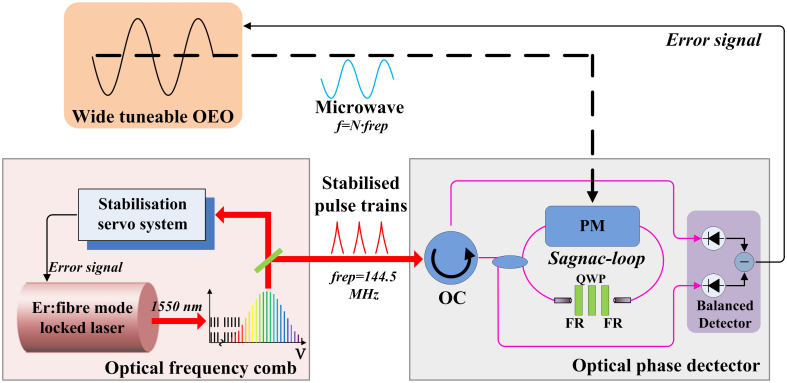
Schematic for wideband microwave extraction by synchronizing OEO with an OFC using an optical phase detector. OC: optical circulator; PM: phase modulator; FR: Faraday rotator; QWP: quarter-wave plate; *f*_rep_: repetition frequency. A microwave generated from a widely tunable OEO is synchronised with the harmonics of an OFC by an optical phase detector. The feedback error signal from the optical-microwave phase detector is used to finely adjust the OEO and to lock the OEO to the harmonics of the OFC. Extremely stable microwaves are extracted from the OFC at an arbitrary harmonic frequency.

**Figure 2:**
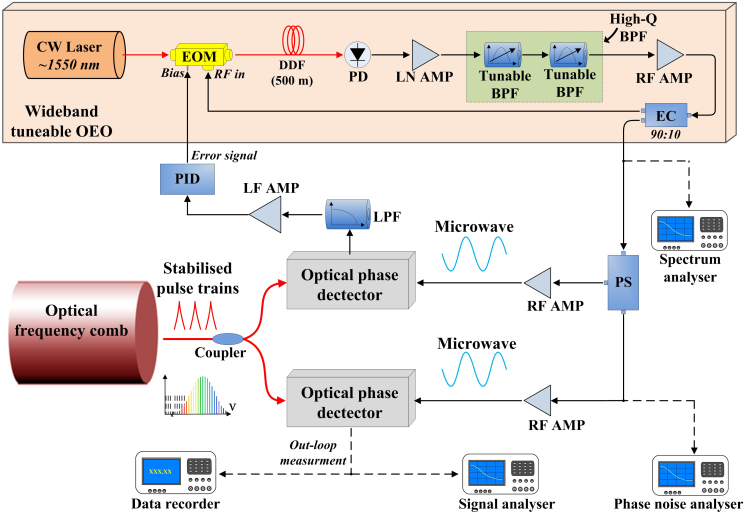
Experimental setup of the synchronisation and measurement system. EOM: electro-optic modulator; DDF: dispersion-decreasing fibre; PD: photodetector, LN AMP: low-noise amplifier; BPF: bandpass filter; LPF: low-pass filter; RF: radio frequency; EC: electronic coupler; PS: power splitter; PID: proportion integration differentiation. Two optical phase detectors are used in our synchronisation system. One detector is used to detect the phase difference between the OEO and the pulse trains to generate the in-loop phase error. This error signal is low-pass filtered, amplified, PID-regulated, and then fed back to the EOM to lock the OEO to the pulse train. The other optical phase detector is used to generate the out-of-loop phase error for measurements of the out-of-loop residual phase noise, phase drift, and relative frequency instability.

**Figure 3:**
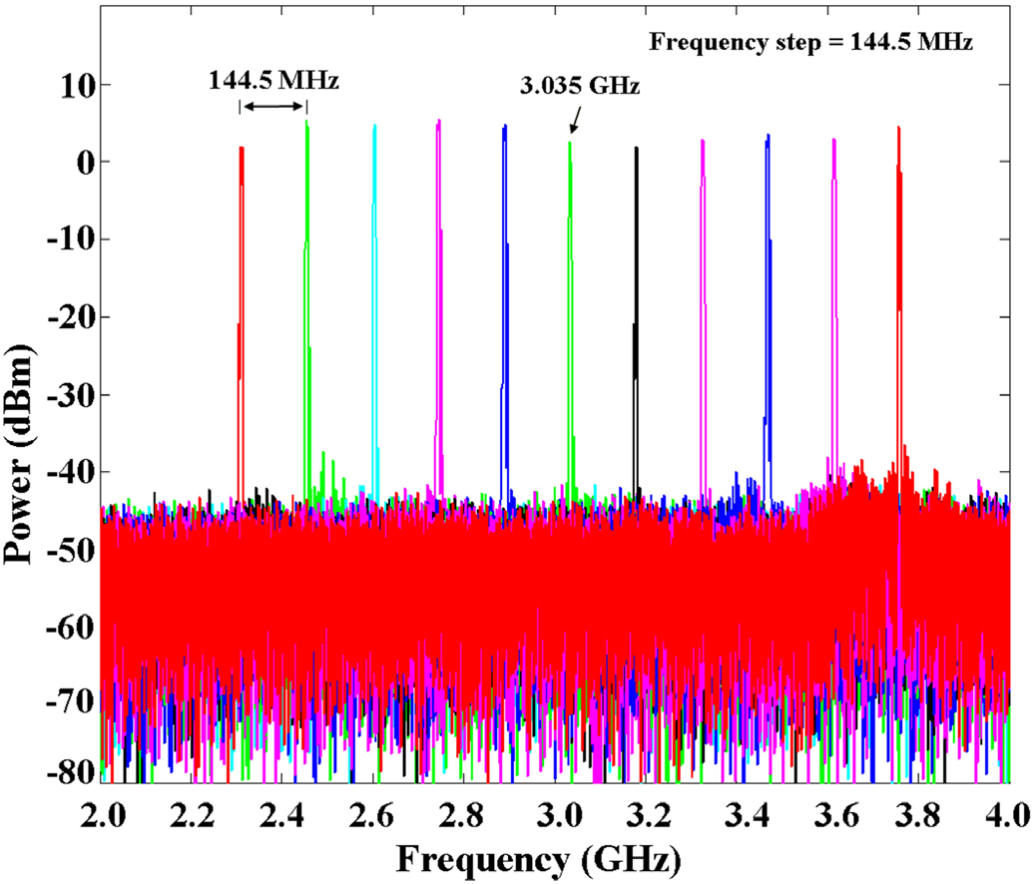
Spectra of the generated microwaves from the OEO at different harmonic frequencies from 2 to 4 GHz. By adjusting the centre frequency of the electronic filter group, a widely tunable free-running OEO range of 2 to 4 GHz is achieved with a fine frequency step of 144.5 MHz. The frequencies of the microwaves match the harmonics of the OFC.

**Figure 4:**
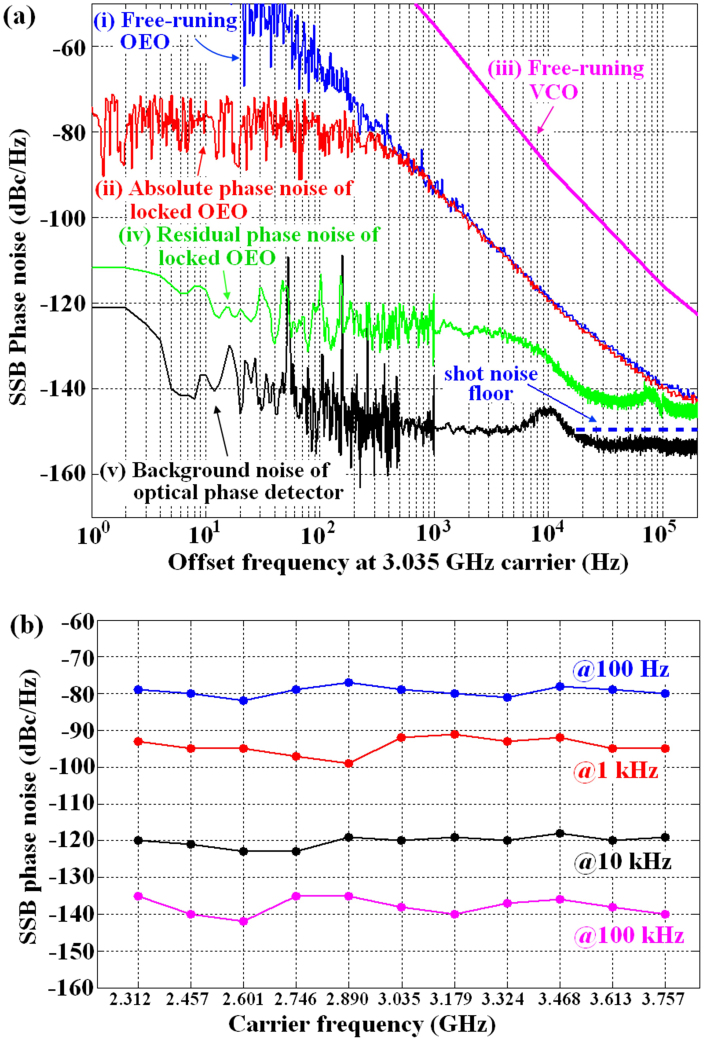
SSB phase noise measurement results (with a measurement bandwidth of 500 kHz). (a), (i) and (ii) Absolute phase noise of the free-running and locked OEO, respectively. (iii) Absolute phase noise of a commercial VCO. (iii) Out-of-loop residual phase noise of the OEO locked to the OFC (integrated rms timing jitter approximately 2.2 fs [1 Hz–100 kHz]). (iv) Background noise of the optical phase detector when the microwave signal is not applied. (b), The absolute phase noise of the locked OEO at eleven discrete carrier frequencies.

**Figure 5:**
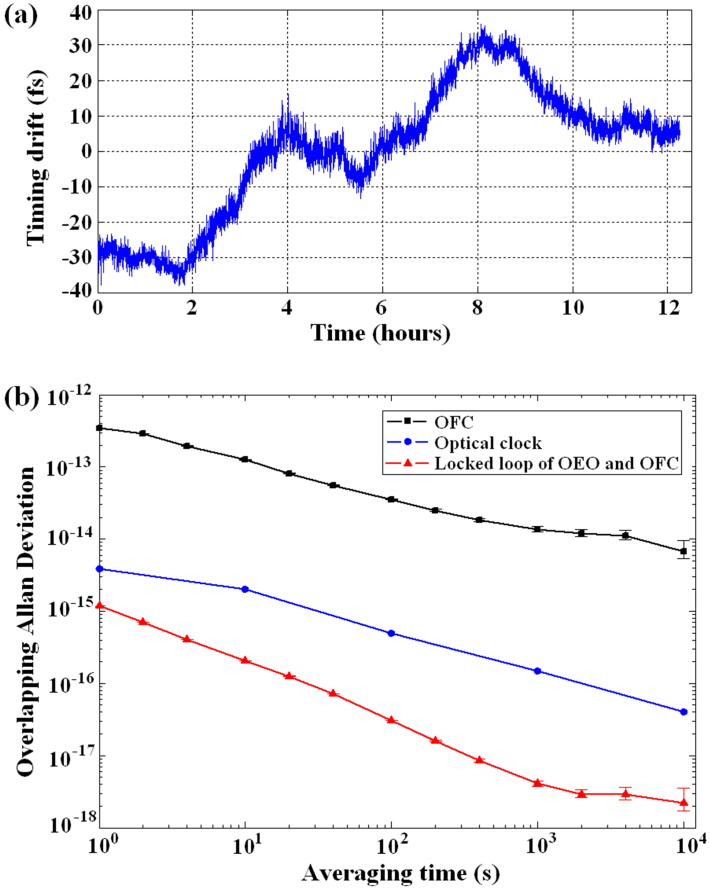
Temporal record of residual timing drift and fractional frequency instability (for a measurement bandwidth of 100 Hz). (a), Timing drift records for the locked OEO. The measurement has a duration of approximately 12 hours and results in an 18-fs (rms) drift. (b), The frequency instabilities of the H-master-stabilised OFC and an optical clock; the fractional frequency instability for the locked loop between the OEO and the OFC. This instability is calculated as the overlapped Allan deviation from the timing drift records.

The details of the optical phase detector are described in Jung and Kim^15^. The optical phase detector is used to implement an optoelectronic phase-locked loop that locks the zero-crossings of a microwave signal to the optical pulse train. The optical phase detector illustrated in Figure 1 consists of an optical circulator, a 50:50 fibre coupler, a unidirectional phase modulator, and a nonreciprocal quarter-wave bias unit with two Faraday rotators and a quarter-wave plate. A polarisation-maintaining (PM) fibre is used to link these optical units and to implement a Sagnac loop. The pulse train from the OFC is applied to the phase modulator driven by the microwave signal (generated from the OEO). At the output of the Sagnac loop, the output pulse train is modulated with an amplitude proportional to the phase error between the pulse train and the microwave signal. The power difference between the two outputs of the Sagnac loop is proportional to the phase error between the optical pulse train and the microwave signal. A balanced photodetector is used to detect the power difference and to generate an error voltage signal that precisely reflects the optical-microwave phase difference. The key issue in the implementation of the optical phase detector is detecting the phase error between the optical pulse train and the microwave signal in the optical domain before the photodetection is involved.

### Stabilised Er-doped fibre OFC

The stabilisation of the OFC is described in Washburn et al.^24^. Here, we offer a general description of the stabilised OFC system. A passively polarisation additive-pulse mode-locked (P-APM) Er-doped MLL is used as the OFC source. This Er-fibre MLL, similar to that in Chen et al.^25^, has a fundamental repetition rate of 144.5 MHz. To realise a highly stable OFC, the MLL's 6^th^ harmonic at 867 MHz was directly locked to an H-master (MHM-2010)^26^ referenced microwave synthesiser (Agilent, E8257D), and the carrier-envelope offset frequency of the MLL was also locked to the same H-master. Based on the locking technique, a stable Er-fibre-based OFC with a wavelength ranging from 1510 nm to 1610 nm, centred at 1560 nm is achieved. This OFC has a pulse width of approximately 150 fs, an average optical power of approximately 35 mW, and a repetition rate of 144.5 MHz.

### Extraction and measurement system

A low-noise PIN PD is utilised in our OEO system to extract the high-purity microwave signal from the optoelectronic loop. The PD, a pair of jointly packaged, fibre-coupled, 5-GHz, InGaAs PIN PDs (50-Ω terminated, +12-V bias), is used to convert the optical power of the OEO to microwaves. This PD has an active diameter of 75 μm, a responsivity of 0.85 A/W at 1550 nm, and a return loss of −55 dB. Light near 1550 nm was coupled to the PD after a long delay fibre cable and was converted into a high-purity microwave signal. The microwave was split into two parts with unequal powers in an electronic coupler. The part with the lower power of approximately 3 dBm was used as the output of the OEO to achieve synchronisation.

For spectrum analysis, the microwave generated from the OEO was bandpass filtered, and directly input to spectrum analyser (Agilent, N9320A). For phase noise measurements, a phase noise analyser (Agilent, E5052B) was used to measure the absolute phase noise of the OEO. In addition, to estimate the synchronization performance, we measured and recorded an out-of-loop phase errors signal via a signal analyser (Agilent, EXA N9010A) and a digital storage oscilloscope (Tektronix, MSO4104), for calculating the phase drift and residual phase noise. The relative frequency instability of the locked loop was calculated from the time series of recorded data. The frequency instability and phase noise measurements are similar to those in obtained in the studies of Kim et al., Jung and Kim, and Hajimiri and Lee^13^,^15^,^16^, where further details can be found.

## Author Contributions

J.Y.Z. and D.H. developed the concept. D.H. and J.T.W. designed the optical frequency comb. X.P.X. and Z.Y.C. designed the optoelectronic oscillator. D.H. and Y.L.Z. developed the optical phase detector and the locking servo system. J.Y.Z. provided technical guidance for the experimental setup. D.H. conducted the experiments and collected the data. All authors performed experiments and contributed to the final manuscript.
